# Interpretable Machine Learning for Osteopenia Detection: A Proof-of-Concept Study Using Bioelectrical Impedance in Perimenopausal Women

**DOI:** 10.3390/jfmk10030262

**Published:** 2025-07-11

**Authors:** Dimitrios Balampanos, Christos Kokkotis, Theodoros Stampoulis, Alexandra Avloniti, Dimitrios Pantazis, Maria Protopapa, Nikolaos-Orestis Retzepis, Maria Emmanouilidou, Panagiotis Aggelakis, Nikolaos Zaras, Maria Michalopoulou, Athanasios Chatzinikolaou

**Affiliations:** 1 Department of Physical Education and Sport Science, School of Physical Education, Sport Science and Occupational Therapy, Democritus University of Thrace, 69100 Komotini, Greece; dimibala10@phyed.duth.gr (D.B.); ckokkoti@affil.duth.gr (C.K.); tstampou@phyed.duth.gr (T.S.); alavloni@phyed.duth.gr (A.A.); dpantazi@phyed.duth.gr (D.P.); mprotopa@phyed.duth.gr (M.P.); nretzepi@phyed.duth.gr (N.-O.R.); maemmano@phyed.duth.gr (M.E.); pangelak@phyed.duth.gr (P.A.); nzaras@phyed.duth.gr (N.Z.); michal@phyed.duth.gr (M.M.); 2 Department of Life Sciences, School of Life and Health Sciences, University of Nicosia, 2417 Nicosia, Cyprus

**Keywords:** public health screening, perimenopause, preventive medicine

## Abstract

**Objectives:** The early detection of low bone mineral density (BMD) is essential for preventing osteoporosis and related complications. While dual-energy X-ray absorptiometry (DXA) remains the gold standard for diagnosis, its cost and limited availability restrict its use in large-scale screening. This study investigated whether raw bioelectrical impedance analysis (BIA) data combined with explainable machine learning (ML) models could accurately classify osteopenia in women aged 40 to 55. **Methods:** In a cross-sectional design, 138 women underwent same-day BIA and DXA assessments. Participants were categorized as osteopenic (T-score between −1.0 and −2.5; n = 33) or normal (T-score ≥ −1.0) based on DXA results. Overall, 24.1% of the sample were classified as osteopenic, and 32.85% were postmenopausal. Raw BIA outputs were used as input features, including impedance values, phase angles, and segmental tissue parameters. A sequential forward feature selection (SFFS) algorithm was employed to optimize input dimensionality. Four ML classifiers were trained using stratified five-fold cross-validation, and SHapley Additive exPlanations (SHAP) were applied to interpret feature contributions. **Results:** The neural network (NN) model achieved the highest classification accuracy (92.12%) using 34 selected features, including raw impedance measurements, derived body composition indices such as regional lean mass estimates and the edema index, as well as a limited number of categorical variables, including self-reported physical activity status. SHAP analysis identified muscle mass indices and fluid distribution metrics, features previously associated with bone health, as the most influential predictors in the current model. Other classifiers performed comparably but with lower precision or interpretability. **Conclusions:** ML models based on raw BIA data can classify osteopenia with high accuracy and clinical transparency. This approach provides a cost-effective and interpretable alternative for the early identification of individuals at risk for low BMD in resource-limited or primary care settings.

## 1. Introduction

Osteoporosis is a chronic skeletal disorder marked by reduced bone mineral density (BMD) and deterioration of bone microarchitecture, leading to increased susceptibility to low-trauma fractures [1,2]. It primarily affects older adults, particularly postmenopausal women, due to the decline in estrogen levels that accelerates bone resorption and impairs bone formation [3,4]. Although the disease is often asymptomatic in its early stages, it can result in fragility fractures that substantially impact mobility, independence, and overall quality of life [5]. These fractures most frequently involve the hip and vertebrae, clinically relevant regions, and are commonly assessed through BMD measurements at the proximal femur, which strongly predict future fracture risk [6]. Despite a well-defined risk profile, osteoporosis is often diagnosed only after menopause, typically following a clinical fracture. At that point, bone loss is already advanced, reducing the effectiveness of preventive measures [7,8].

Dual-energy X-ray absorptiometry (DXA) remains the gold standard for assessing bone mineral density (BMD), and is most commonly utilized in postmenopausal populations to diagnose osteoporosis. However, its application during the menopausal transition could enable the earlier identification of individuals at elevated risk and allow for timely, preventive interventions. Importantly, modern DXA systems are not limited to skeletal assessment; they also provide comprehensive body composition data, including estimates of lean mass, fat mass, and appendicular skeletal muscle mass—metrics increasingly recognized as integral to bone health [9,10,11,12]. Body composition is typically described as comprising three fundamental components: lean tissue mass, fat mass, and bone mineral content [13,14]. The distribution and interaction of these compartments play a key role in musculoskeletal health. Mechanical loading from skeletal muscle promotes bone remodeling [15], while excess adiposity may impair bone integrity through inflammatory and endocrine pathways [16].

Mechanical loading from skeletal muscle stimulates bone remodeling, while excessive adiposity may have adverse effects through inflammatory and hormonal mechanisms. As a result, understanding soft tissue distribution offers additional insight into skeletal integrity, particularly in aging populations where sarcopenia and increased fat mass frequently coexist [17,18]. However, DXA’s body composition capabilities are often underused in routine practice. This underutilization stems not from technological limitations but from practical barriers, including cost, limited availability, and the need for trained personnel. These factors constrain the feasibility of widespread DXA-based screening, especially in primary care or community settings where early risk identification is most valuable.

In light of the practical limitations associated with DXA-based assessments—namely, cost, limited availability, and the requirement for specialized personnel—there is growing interest in more accessible methods for estimating body composition. Bioelectrical impedance analysis (BIA), initially developed for this purpose, offers several advantages, including portability, low cost, and ease of use [19,20]. Although BIA does not measure bone mineral density and is not intended as a diagnostic tool for skeletal disorders, it reliably estimates fat mass and lean mass, two components increasingly associated with bone strength and fracture risk [21,22]. In addition, BIA-based algorithms can provide reasonably accurate estimates of basal metabolic rate (BMR), further supporting their utility in clinical and preventive health contexts [23].

In this context, BIA serves as a practical adjunct in musculoskeletal health monitoring, especially in community or primary care settings where DXA may not be available. Several studies have reported strong correlations between BIA- and DXA-derived measurements of body composition across various populations [24,25]. Moreover, emerging evidence suggests that BIA-derived indices, such as the skeletal muscle index (SMI), may help identify individuals at risk for sarcopenia and associated bone fragility [26,27]. In the present study, BIA-derived measurements were directly compared with DXA values, demonstrating high agreement across key parameters and reinforcing the feasibility of using BIA in population-level screening strategies.

Integrating accessible methods, such as bioelectrical impedance analysis (BIA), into preventive care is both clinically valuable and socioeconomically significant. Fragility fractures represent a major public health burden, accounting for millions of hospitalizations, long-term care placements, and productivity losses each year. In Europe alone, the economic burden of incidents and prior fragility fractures was estimated at EUR 37 billion, with incident fractures representing 66% of this cost [28]. Beyond the financial strain, the human cost of fractures is severe, particularly for hip fractures, which carry a high mortality risk. During the first year of follow-up, individuals with a hip fracture had an adjusted hazard ratio of 3.2 (95% CI 1.4–7.4) for increased mortality. Vertebral fractures also contribute to higher mortality, with those experiencing a vertebral fracture in the second year having an adjusted hazard ratio of 2.7 (95% CI 1.1–6.6). Among women, vertebral fractures in the first or second year were linked to increased mortality, with hazard ratios of 3.7 (95% CI 1.1–12.8) and 3.2 (95% CI 1.2–8.1), respectively [29]. These outcomes disproportionately affect vulnerable populations, often leading to a permanent loss of independence and increased reliance on healthcare and social support systems [30]. Shifting toward earlier, community-level risk detection—through scalable tools like BIA—could significantly reduce fracture incidence and the downstream consequences of osteoporotic complications.

Recent machine learning (ML) advances allow leveraging BIA-derived data to predict osteopenia and osteoporosis risk [31,32,33]. Unlike traditional statistical approaches, ML techniques can model complex, non-linear relationships among multiple variables [34,35,36]. Shim et al. [37] developed and validated several ML models for predicting osteoporosis risk using demographic and health survey data from a large Korean cohort, with their best-performing model achieving an AUROC of 0.743. In another study, Lim et al. [33] applied ML and radiomics to abdominal–pelvic CT scans, reporting exceptionally high AUROC values above 0.95, but at the cost of requiring high-resolution imaging and clinical infrastructure. When combined with explainable artificial intelligence (XAI) frameworks [38,39], such models offer both predictive power and interpretability, enabling clinicians and researchers to understand how specific body composition features contribute to low BMD. Kim et al. [37] used XAI to identify patients with osteopenia and sarcopenia in daily life. Specifically, the XGBoost classifier achieved an accuracy of 88.69% for osteopenia, while the random forest (RF) classifier reached 93.75% for sarcopenia.

This study aims to develop and validate interpretable ML models to predict osteopenia in women aged 40 to 55 years, using raw BIA data as input. The specific objectives are as follows: (i) identify the most significant body composition predictors of low BMD across different levels of physical activity; (ii) evaluate the predictive performance and transparency of various XAI algorithms; and (iii) explore the potential of these models to support early, accessible screening strategies in clinical and community settings. Integrating body composition analysis with advanced analytics may provide a scalable alternative to DXA, enabling timely interventions to maintain bone health during midlife.

To guide the development and evaluation of the machine learning models, two primary hypotheses were formulated. The first hypothesis proposed that raw outputs from bioelectrical impedance analysis (BIA), including impedance values, phase angles, and segmental tissue composition, would be sufficient to classify osteopenia in women aged 40 to 55 years without the need for derived indices or imaging-based inputs. The second hypothesis posited that incorporating categorical variables such as chronological age, menopausal status, and history of sports participation would improve both model performance and interpretability by introducing clinically relevant demographic and behavioral factors related to bone health.

## 2. Materials and Methods

### 2.1. Study Design

This cross-sectional study aimed to develop and assess explainable ML models for classifying osteopenia in women aged 40 to 55 years. It utilized raw BIA data, integrating body composition metrics—such as lean and fat mass—with essential categorical variables, including age, menopausal status, history of sports participation, and self-reported physical activity level. These factors are well-recognized determinants of bone strength and remodeling. These variables, all readily obtainable in clinical or community settings, were employed to predict BMD status as an indicator of osteoporosis risk. The objective was to determine whether this approach could serve as a cost-effective and accessible alternative to DXA for early screening.

All assessments were conducted during a single laboratory visit at the Department of Physical Education and Sport Science, Democritus University of Thrace. Participants were instructed to arrive euhydrated, having fasted overnight for at least 8 h, and to abstain from alcohol, caffeine, and strenuous physical activity for a minimum of 48 h prior to testing. The testing sequence included anthropometric measurements, BIA for the estimation of body composition, and DXA to quantify BMD. To assess the feasibility of using BIA-derived metrics in place of DXA, additional analyses were conducted to evaluate the agreement between BIA and DXA estimates of fat mass and fat-free mass. DXA scans targeted the hip region, given its clinical relevance in predicting fracture risk. Before all assessments, participants removed metallic items to eliminate measurement interference.

Categorical variables obtained through structured interviews—such as age, menopausal status, physical activity level, and history of sports participation—were included in the dataset alongside raw bioelectrical impedance metrics. These variables served as predictors in supervised classification models developed to distinguish between osteopenic and non-osteopenic women. Osteopenia status was determined using DXA-derived BMD at the total hip, a site with high predictive value for osteoporotic fractures. Following World Health Organization (WHO) criteria, women with T-scores between −1.0 and −2.5 were classified as osteopenic, while those with T-scores above −1.0 were considered non-osteopenic [40]. Individuals with T-scores ≤ –2.5 were excluded to ensure the models targeted early stage bone loss rather than established osteoporosis. This binary classification allowed the development of interpretable machine learning models focused on identifying subclinical bone deterioration in a community-dwelling female population.

The dataset used for model development consisted of 226 predictors (Table S1), organized into two principal categories: (i) raw electrical parameters and device-derived body composition estimates obtained from the multi-frequency BIA device (e.g., impedance values, phase angles, segmental lean and fat mass), and (ii) categorical variables collected through structured interviews, including age, menopausal status, sports participation history, and self-reported physical activity level. These inputs were used to train supervised ML models for classifying osteopenic and non-osteopenic individuals. Given the moderate class imbalance, data preprocessing included cross-validation and appropriate resampling methods to ensure robust model performance. The study received ethical approval from the Institutional Ethics Committee of Democritus University of Thrace (reference number: DUTH/EHDE/62808/581), and all participants provided written informed consent in accordance with the Declaration of Helsinki.

### 2.2. Participants

The study involved 138 women aged between 40 and 55 years. Participants were recruited through public advertisements and community outreach efforts and selected based on predefined eligibility criteria.

Inclusion criteria stipulated that participants must fall within the specified age range and be free from metabolic disorders that could affect bone health or fluid balance. Exclusion criteria encompassed factors that might interfere with measurement accuracy or confound bone-related outcomes. Participants were excluded if they had pacemakers, metallic implants, or other electronic medical devices that could disrupt BIA or DXA assessments. Women with metabolic disorders influencing bone health or fluid balance, such as thyroid dysfunction or chronic kidney disease, were also excluded. Additional exclusions included current pregnancy, active menstruation on the testing day, recent use of medications known to affect bone metabolism or body composition, and any major musculoskeletal injuries within the previous six months. Individuals with a history of osteoporosis-related fractures, bone diseases, or significant anatomical alterations, such as limb loss, were also excluded to ensure the consistency and validity of the measurements.

### 2.3. Procedures

#### 2.3.1. Interview and Categorical Data Collection

Structured interviews were administered to collect essential participant information, including age, self-reported physical activity level, menopausal status, and history of sports participation. Menopausal status was defined as the absence of menstruation for at least 12 consecutive months. Interviews were conducted by trained personnel using a standardized script to ensure consistency and reliability. All categorical variables were subsequently coded as binary values (0 or 1) to facilitate integration into the supervised machine learning models. These included menopausal status (pre- vs. postmenopausal), a history of sports participation (yes/no), and current physical activity engagement (active/inactive). The categorical variables were treated as predictors alongside the BIA-derived body composition metrics.

#### 2.3.2. Height Measurement

Standing height was measured to the nearest millimeter using a mechanical stadiometer (Seca, Germany). Participants were barefoot and positioned with their heels together, knees extended, and backs straight, maintaining a neutral head alignment. The headpiece was gently lowered to rest on the crown of the head, taking into account the compression of any hair. Two measurements were taken, and the average of these readings was recorded for analysis. The same trained researcher conducted all assessments to ensure consistency and minimize inter-rater variability.

#### 2.3.3. Body Composition Assessment

Body composition was evaluated using a multi-frequency BIA device (MA801, Charder, Taiwan, China), which performs a comprehensive full-body scan through segmental analysis at five different frequencies (5, 50, 100, 250, and 500 kHz). All measurements were taken meticulously in accordance with the manufacturer’s guidelines. Participants were instructed to remove their shoes, socks, and any metallic objects prior to the assessment. Measurements were taken with participants standing barefoot on the designated foot electrodes while holding the hand grips to ensure optimal sensor contact. To minimize variability related to fluid levels and enhance consistency, BIA measurements were conducted before any other assessments and always under fasting or standardized pre-assessment conditions. Each participant underwent two consecutive BIA measurements to ensure the accuracy and repeatability of the results. If the two measurements differed by more than 0.5 kg in total body weight or if discrepancies in body composition parameters exceeded the device’s acceptable limits, a third measurement was conducted, with the two most consistent results being averaged. All assessments were conducted at approximately the same time of day for all participants to reduce circadian influences on fluid distribution.

##### Validation of BIA Against DXA

To evaluate agreement between BIA and DXA estimates of fat mass (FM) and fat-free mass (FFM), a multi-method comparison was conducted. Pearson correlation coefficients (r) were used to assess linear association, while paired samples t-tests evaluated mean differences, conditional on normality of the differences. The mean percentage error, defined as the signed difference divided by the DXA value and multiplied by 100, was used to quantify the relative bias. Bland–Altman analysis was performed to assess systematic bias and 95% limits of agreement (LoA), with corresponding confidence intervals reported for both. Proportional bias was examined visually and tested via the linear regression of the differences on the mean values. Intraclass correlation coefficients (ICC) were calculated using a two-way mixed-effects model for single measures with absolute agreement, and reported with 95% confidence intervals to assess consistency.

#### 2.3.4. Bone Health Assessment

Bone mineral density was assessed using a GE Healthcare Lunar DPX NT Bone Densitometer (2012) under standardized laboratory conditions [41]. Participants removed all metallic objects to avoid scan interference. Dual femur scans were conducted, focusing specifically on the proximal femur region due to its clinical relevance in osteoporosis-related fracture risk. The procedure involved minimal radiation exposure (0.01–0.03 mSv), which was substantially lower than that of a standard chest X-ray.

Participants were positioned following updated guidelines [42]. Arms and legs were kept straight and parallel, with the toes pointing upwards. A foot positioning device secured with a strap induced ~15–20° of internal hip rotation, aligning the femoral neck parallel to the table and minimizing anatomical distortion. Positioning was visually inspected, and soft tissue artifacts were excluded when necessary.

Regions of interest (ROIs) included the femoral neck, upper and lower femoral neck, Ward’s region, greater trochanter, femoral shaft, and total hip. Automated segmentation was performed using enCORE version 14.10.022 software, with manual adjustments as needed to ensure anatomical accuracy. Osteopenia classification was based on T-scores derived from the total hip. According to WHO criteria, participants with T-scores between −1.0 and −2.5 were classified as osteopenic. Those with T-scores ≥ −1.0 were considered to have normal bone mass, while individuals with T-scores ≤ −2.5 (within the osteoporotic range) were excluded from further analysis, allowing the model to focus on early stage bone loss.

### 2.4. Machine Learning Workflow

Data Preprocessing. A structured preprocessing approach was employed to enhance data quality and ensure consistency across various study phases. Missing data, which were limited to continuous variables and accounted for no more than 5% of the dataset, were handled using the mode imputation strategy. Furthermore, all features were standardized using the StandardScaler library, which normalizes the data by removing the mean and scaling it to unit variance. This preprocessing step was uniformly applied throughout the feature selection (FS) and ML model training phases, ensuring data representation consistency and optimizing the learning process.

Feature Selection. To identify the most relevant predictors for classification from a pool of 226 features (predictors), we employed Sequential Forward Feature Selection (SFFS) in conjunction with 5-fold Stratified Cross-Validation. SFFS is an iterative approach that incrementally builds an optimal feature subset by sequentially adding the feature that maximizes model performance at each step. Stratified cross-validation ensures that the class distribution is maintained in each fold, which is crucial for imbalanced datasets. The SFFS method offers multiple advantages: it enhances model predictive accuracy, systematically selects the most influential variables, reduces model complexity and the risk of overfitting, and ultimately improves model interpretability and generalizability to new data.

Machine Learning Model Training. Our study utilized four different ML algorithms—Extreme Gradient Boosting (XGBoost) [43], Support Vector Machines (SVMs) [44], Logistic Regression (LR) [45], and Neural Networks (NNs) [46]—for binary classification. Adopting a multi-algorithmic approach enabled us to comprehensively evaluate the robustness of our models by comparing their performance, thereby mitigating potential biases associated with any single algorithm and gaining insights into the impact of different features on classification outcomes. Given the absence of a universally optimal ML algorithm for this specific task, using multiple classifiers strengthens our findings’ reliability and generalizability of our findings. The FS process was conducted separately for each algorithm to accommodate the unique predictive power interactions of each classifier with the selected features. To further enhance model performance and mitigate overfitting, we employed a 5-fold stratified cross-validation strategy. Within each training fold, we applied the Synthetic Minority Over-sampling Technique (SMOTE) to address class imbalance, ensuring that oversampling was confined to the training data only, thereby preventing data leakage into the validation folds. Additionally, hyperparameter tuning was performed within the training set using a nested 5-fold stratified cross-validation to identify the optimal configurations for each classifier. This comprehensive methodology enabled us to extract meaningful insights and achieve high predictive accuracy, underscoring the adaptability and efficacy of these ML techniques.

Performance Evaluation. A diverse set of evaluation metrics was employed to rigorously assess the predictive performance of the ML models. Accuracy, defined as the proportion of correctly classified instances among the total samples, served as a fundamental performance indicator. Precision, representing the proportion of correctly identified positive cases among all predicted positives, was used to assess the reliability of positive predictions. Recall (sensitivity) measured the model’s capability to identify all relevant positive cases, capturing the proportion of actual positives correctly classified. The F1-score, a harmonic mean of precision and recall, provided a balanced measure of performance. Additionally, the area under the receiver operating characteristic (ROC) curve (AUC-ROC) was used to quantify the degree of separability between classes. At the same time, the mean normalized confusion matrix was analyzed to understand the distribution of classification errors across categories.

Model Interpretability. To gain a deeper understanding of how individual features contributed to the model’s predictions, we utilized SHapley Additive exPlanations (SHAP) [47]. SHAP values provide a consistent and interpretable measure of feature importance by attributing each prediction to the contribution of individual features. Rooted in cooperative game theory, the SHAP framework enables a transparent interpretation of complex model behaviors. In our analysis, SHAP was applied to the standardized input data used during model training to maintain consistency in feature representation. This approach allowed us to identify the most influential predictors and understand their directional impact on the model’s decision-making process [48].

Implementation. All ML model development, training, and evaluation procedures were executed using Python 3.9. The Scikit-learn library (https://scikit-learn.org/, accessed on 20 March 2025) served as the primary framework for implementing ML algorithms and preprocessing techniques, ensuring reproducibility and efficiency in our analytical workflow.

## 3. Results

### 3.1. Validity of the BIA Device in the Study’s Population

**Figure 1:**
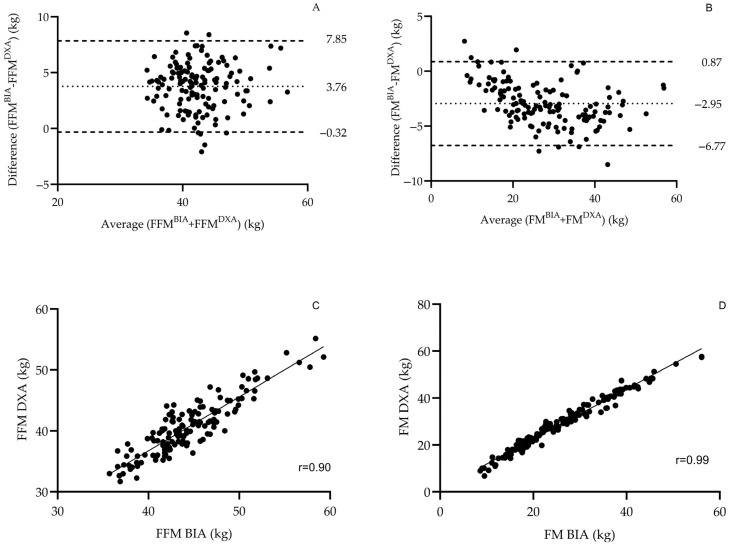
Agreement between DXA and BIA measurements for fat-free mass (FFM) and fat mass (FM). Panels (**A**,**B**) show Bland–Altman plots comparing FFM and FM values obtained from dual-energy X-ray absorptiometry (DXA) and bioelectrical impedance analysis (BIA), illustrating the mean difference and limits of agreement. Panels (**C**,**D**) display scatterplots with linear regression lines showing the strong correlations between the two methods for FFM and FM, respectively, across the total sample.

To validate the use of BIA as an input source, agreement between BIA and DXA measurements was evaluated in the full sample. Strong correlations were observed between BIA- and DXA-derived FM (r = 0.99, *p* < 0.001) and FFM (r = 0.90, *p* < 0.001). However, BIA underestimated FM by approximately 2.95 kg and overestimated FFM by about 3.76 kg, both differences reaching statistical significance (*p* < 0.001). Bland–Altman plots and scatterplots illustrating these relationships are presented in Figure 1.

#### Participant Characteristics

Table 1 presents the descriptive characteristics of the study population, stratified by osteopenia status. Significant differences were observed between osteopenic and non-osteopenic participants across several anthropometric and body composition variables.

### 3.2. Performance Metrics

Table 2 summarizes the testing performance metrics of the employed ML classifiers, including average accuracy, recall, precision, F1-score, and AUC-ROC. Each classifier was independently applied following the FS process, ensuring optimized performance for predicting BMD levels.

The NN classifier emerged as the best-performing model, achieving an accuracy of 86.55%, a recall of 76.48%, a precision of 84.06%, and an F1-score of 79.77%, using 17 selected features. It also attained the highest ROC AUC (85.90%), highlighting its strong and balanced discriminative ability across metrics. XGBoost also demonstrated robust performance, with the highest accuracy (88.48%) among all models and a competitive F1-score of 77.69% and ROC AUC of 85.25%, making it a reliable alternative. Logistic Regression achieved high precision (95.01%) and strong accuracy (87.01%), but its recall was substantially lower (49.06%), indicating a tendency to miss positive cases. Support Vector Machine showed the poorest recall (0.00%) and the lowest ROC AUC (47.50%), indicating limited ability to detect true positives. However, it achieved a high precision of 89.53%, suggesting that its few positive predictions were mostly correct, albeit at the expense of high false-negative rates.

**Figure 2:**
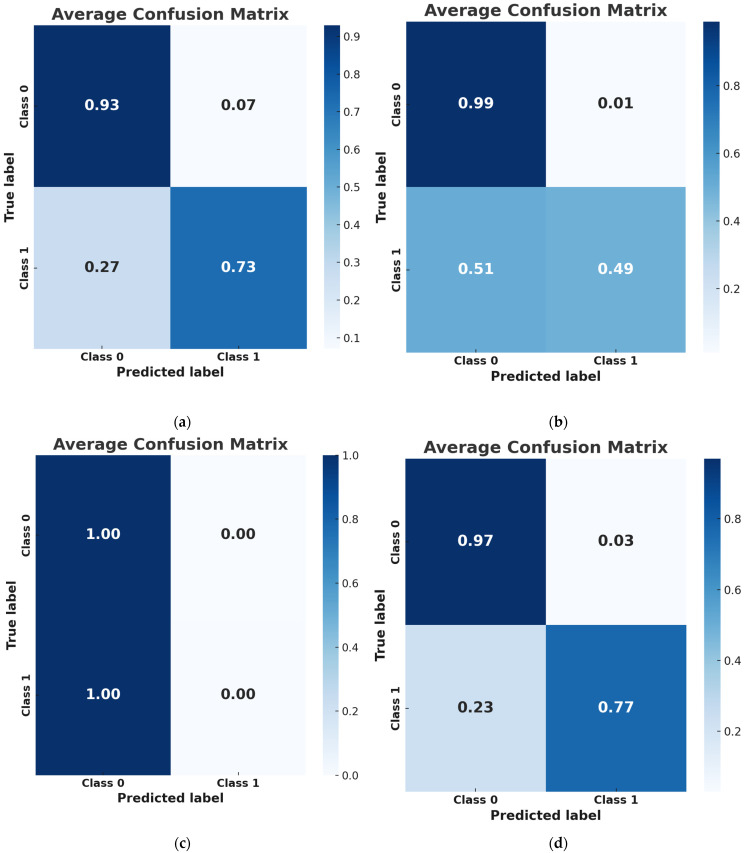
Confusion matrices for the machine learning (ML) classifiers used in the study. (**a**–**d**) represent the performance of the (**a**) XGBoost, (**b**) LR, (**c**) SVM, and (**d**) NN classifiers, respectively. Each matrix displays the number of true positives, true negatives, false positives, and false negatives, providing a visual summary of each model’s ability to correctly classify cases and non-cases of osteopenia. Sensitivity and specificity values are derived from these matrices.

To provide further insights into classifier performance, Figure 2 presents the confusion matrices for all models, illustrating their ability to classify positive and negative cases correctly. The NN classifier demonstrated an actual positive rate (sensitivity) of 76.67% and an actual negative rate (specificity) of 97.14%, reinforcing its capability as a reliable screening tool for assessing osteoporosis risk.

### 3.3. Feature Selection

Table 3 presents the most informative predictors identified for the binary classification task using the SFFS technique. The NN model, which achieved the highest accuracy, utilized 34 key predictors to differentiate individuals based on BMD levels.

### 3.4. Interpretation

**Figure 3:**
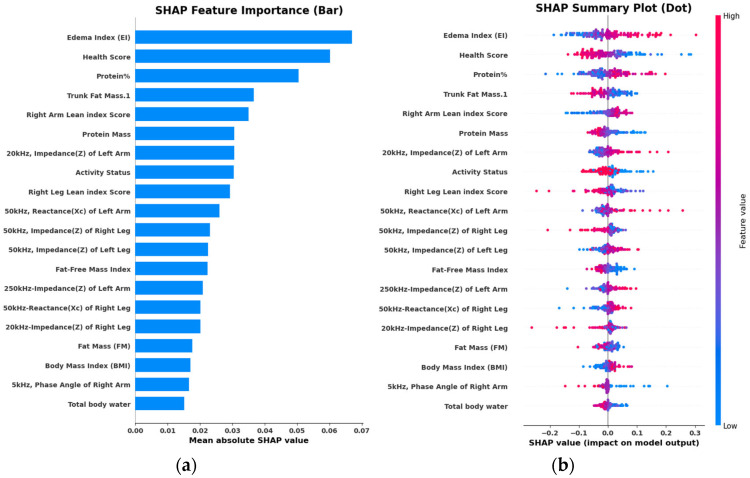
SHAP analysis of feature importance based on the NN classifier. Figure (**a**) shows the mean absolute SHAP values for each predictor, indicating their overall contribution to the model’s output. Figure (**b**) presents the SHAP summary plot, which displays both the magnitude and direction of each feature’s impact on individual predictions across the dataset. Higher SHAP values correspond to a stronger influence on model decisions, with color gradients indicating the magnitude of feature values.

To interpret the NN model output, we employed the SHAP model. SHAP values provide a unified measure of feature (predictor) importance by attributing the contribution of each predictor to the model’s predictions. Figure 3a presents the mean SHAP values for each predictor, illustrating their overall impact on BMD classification. Edema Index, physical activity status, hydration status, and reactance (50 kHz) of the whole body were identified as the most influential predictors.

Figure 3b provides a SHAP summary plot that displays the magnitude and direction of each feature’s contribution to model output. Higher values of predictors such as protein mass contributed to a lower probability of osteopenia classification, whereas an elevated Edema Index and protein percentage were associated with increased model-predicted risk. These results highlight the importance of segmental and compositional BIA variables, particularly those reflecting fluid balance and muscle quality, in supporting bone health assessment through interpretable machine learning.

## 4. Discussion

The present study evaluated the performance of explainable machine learning models trained on raw multi-frequency bioelectrical impedance analysis data to classify osteopenia in women aged 40 to 55 years. Two hypotheses were examined: first, that raw impedance-based variables alone could achieve the accurate classification of osteopenia; and second, that the inclusion of categorical variables such as age, menopausal status, and physical activity history would enhance model performance and interpretability. The first hypothesis was supported since models relying solely on raw and device-derived BIA variables achieved robust discriminatory accuracy (AUC = 0.93), underscoring the capacity of impedance-based signals to encapsulate physiologically meaningful correlates of bone status. The second hypothesis received more limited support. Among the categorical predictors, only current physical activity status demonstrated moderate additional value, whereas menopausal status and prior sports participation added little to model performance. This limited enhancement likely reflects both the coarseness and self-reported nature of these variables, as well as the possibility that key biological or behavioral determinants of bone health may already be encoded within the impedance-based features themselves. It is also important to note that the study was exploratory and cross-sectional, which constrains the interpretation of causality and generalizability. Nevertheless, the findings suggest that while categorical context may augment interpretability, raw BIA metrics remain the primary source of diagnostic signal in this application.

To minimize model complexity while retaining performance, dimensionality reduction was conducted using a sequential forward floating selection algorithm, and feature interpretability was achieved via the SHAP framework. Together, these methods preserved both predictive capacity and transparency. Among the most prominent predictors was the Edema Index, a measure representing the ratio of extracellular to total body water [49]. Although not traditionally associated with bone physiology, its predictive utility may be explained by its relationship to hydration status, fluid compartmentalization, and systemic inflammation [50]. These factors are all known to influence skeletal remodeling indirectly [51,52]. Experimental studies, primarily in ex vivo and animal models, suggest that hydration affects bone toughness by altering interactions within the collagen matrix [52]. Elevated extracellular fluid volumes have been linked to chronic, low-grade inflammation, which is a known driver of osteoclast activity and bone loss [51,53,54]. Although the Edema Index is an indirect marker, its prominence in the model supports the relevance of fluid regulation and systemic health in the early pathophysiology of skeletal decline.

Another top-ranking feature was the Health Score, a proprietary index combining several body composition measures, including lean mass index, fat mass index, skeletal muscle index, and phase angle. Although initially designed for motivational or consumer-facing purposes, its high feature importance in the current analysis may reflect the cumulative value of its components. These components are each associated with muscle mass, fat distribution, and cellular integrity, all of which have been linked to bone health [55,56,57]. Notably, none of the constituent metrics showed similar predictive strength when assessed independently. This suggests that the predictive utility of the Health Score may arise from its ability to integrate multiple physiological dimensions into a single index. However, its proprietary nature and lack of algorithmic transparency limit its reproducibility across devices and populations. The score does not represent a clinically validated health index. Although it proved informative in the present modeling context, its generalizability requires further standardization and external verification.

Several standalone variables contributed distinct physiological insights. Total protein mass exhibited a protective association, consistent with the known role of skeletal muscle in supporting bone strength through mechanical loading and myokine signaling [58]. In contrast, protein percentage, often considered a marker of lean tissue proportion [59], was unexpectedly associated with an increased risk of osteopenia. This apparent contradiction may stem from its ratio-based nature; individuals with low absolute muscle mass but minimal fat stores may display high protein percentages that do not reflect adequate muscle volume to support skeletal integrity [60,61,62]. This distinction between density and capacity in physiological measures was further underscored by the absence of fat-free mass among the top predictors. Similarly, higher trunk fat mass was inversely associated with the status of osteopenia. This finding should not be interpreted as evidence that adiposity directly supports bone health. A more cautious interpretation is that increased trunk mass contributes to overall body weight, which may in turn influence skeletal loading, particularly in axial regions [63,64,65]. While adipose tissue is not osteogenic per se, its presence may help offset insufficient mechanical strain in individuals with low lean mass [66,67]. However, the relationship between fat mass and bone health is unlikely to be proportional. Additional adiposity beyond a certain point may confer diminishing mechanical benefit and introduce systemic metabolic alterations that are potentially detrimental to skeletal integrity. Excess fat mass is associated with hormonal imbalance, reduced insulin sensitivity, and disruptions in lipid metabolism, all of which have been implicated in bone remodeling dynamics [53,68,69,70,71,72]. It is also important to note that trunk fat mass, as estimated via bioelectrical impedance analysis, does not distinguish between visceral and subcutaneous compartments and may be influenced by body geometry and tissue conductivity. As such, the observed association may reflect the indirect influence of body mass on structural loading rather than any intrinsic physiological advantage of fat tissue.

Segmental predictors, particularly the lean mass index values of the right arm and right leg, are also ranked highly. This indicates that region-specific muscle mass may offer diagnostic information not captured by whole-body aggregates. However, a closer inspection of the SHAP values revealed an unexpected trend. A higher right arm lean mass index was associated with an increased likelihood of osteopenia. This counterintuitive association may reflect asymmetries in muscle development or disuse-related compensation patterns, especially in a cohort with predominantly sedentary lifestyles. Elevated lean mass index (LMI) in the dominant arm may reflect localized muscular adaptation due to asymmetrical activity patterns, even in the context of reduced whole-body muscle mass [73,74,75]. However, this segmental preservation may not provide sufficient mechanical strain to support bone remodeling at clinically relevant skeletal sites [76]. An alternative explanation involves the misattribution of importance to a regionally preserved variable in individuals with disproportionate lean mass distribution, which may lead to a biased interpretation of features. Disproportionate upper-to-lower body composition has been linked in some populations to increased cardiometabolic and inflammatory risks, although such associations were not directly assessed in the present study [77,78,79]. In contrast, higher LMI in the right leg was associated with more favorable bone status. This supports the interpretation that lower-limb musculature, which is subject to greater mechanical loading through gait and daily movement, may be more relevant for stimulating osteogenesis [80]. The higher specificity of segmental loading may offer a greater osteogenic stimulus, which whole-body LMI could obscure. Whole-body fat-free mass did not appear among the top predictors, suggesting that localized or functional muscle mass may better capture the mechanical strain necessary for bone remodeling. These findings highlight the diagnostic potential of segmental BIA in detecting musculoskeletal asymmetries or localized deficiencies that whole-body metrics may overlook. Future studies should incorporate data on limb dominance, movement patterns, and functional asymmetry to clarify these associations.

In addition to tissue composition, electrical properties across frequency bands also emerged as significant predictors. Segmental impedance and reactance in the 20- to 250-kilohertz range were particularly informative. These values reflect tissue characteristics such as water distribution and membrane function [19,81]. Although these measures are not traditionally used in bone diagnostics, their substantial predictive value supports the hypothesis that muscle quality and cellular integrity may serve as indirect indicators of bone health [82]. Multi-frequency reactance may also reflect mitochondrial and intracellular function, which are increasingly recognized in musculoskeletal health [83,84,85]. Frequency-dependent reactance, in particular, may capture properties related to tissue vitality and fluid balance [19,86,87]. In contrast to the whole-body phase angle, which is widely regarded as a general marker of cellular integrity [88,89] but did not rank prominently in our SHAP-based analysis, segmental and frequency-specific impedance metrics were more effective in capturing localized tissue variations relevant to osteopenia classification. This highlights the diagnostic value of regionally resolved BIA parameters, as whole-body averages may mask meaningful heterogeneity in musculoskeletal tissues within this age-specific cohort.

Although physical activity status did not emerge as a dominant predictor, its inclusion contributed modestly to overall model accuracy. This result is consistent with the known role of mechanical loading from physical activity in promoting bone strength. The limited predictive power of this variable likely reflects its binary categorization and reliance on self-reported data, which reduces measurement sensitivity and accuracy [90]. More detailed and objective activity measures, such as accelerometry, mechanical load indices, or strain profiles, would likely improve model granularity and help clarify the intensity and volume thresholds of movement necessary for bone preservation [91].

When compared with previous machine learning studies, the present model performs favorably. Shim and colleagues, using anthropometric and survey data, reported moderate accuracy in predicting low bone mineral density [37]. In contrast, Lim and colleagues achieved higher accuracy using radiomic features derived from computed tomography scans [33]. The current model attained comparably high classification accuracy using only BIA-derived variables. It required no imaging, biochemical markers, or clinical scoring systems. This highlights the potential of BIA-based models as accessible and scalable tools for osteoporosis screening in primary care or community health settings, especially in contexts where dual-energy X-ray absorptiometry is unavailable or unaffordable.

This study, to our knowledge, is among the first to employ raw, unaggregated BIA inputs for osteopenia classification in midlife women using interpretable ML. This group is often underrepresented in early osteoporosis detection despite strong evidence of rapid bone loss during the perimenopausal transition. The use of SHAP-based explanations strengthens the clinical applicability of this model by providing transparent and physiologically grounded rationales for each prediction. By enabling feature attribution at the individual prediction level, SHAP enhances model transparency and aligns predictions with physiological mechanisms.

Several methodological strengths support the credibility of the results. All data were collected under standardized laboratory conditions. The BIA device’s estimates of fat mass and fat-free mass were internally validated against DXA using correlation analysis in a representative subset of the sample. This confirmed the accuracy of input variables derived from body composition. However, this validation applied only to composition measures, not to bone density outcomes. Feature selection followed a data-driven and conservative procedure to minimize overfitting, and model interpretability was maintained through the use of post hoc explanation tools.

Certain limitations should be considered when interpreting the present findings. As a cross-sectional analysis, the study design does not permit causal inference, and the observed classification accuracy does not imply predictive validity for future bone loss. The sample consisted of 138 Caucasian women aged 40 to 55 from a single research center, representing a demographically specific yet well-defined cohort. Although this limits generalizability, the sample was suitable for the exploratory aims of this pilot investigation and sufficient for developing reliable and interpretable models. Broader applicability to populations such as men, individuals of non-white ethnicity, or those with comorbidities remains to be established. External validation using larger and more diverse cohorts will be necessary to confirm model robustness. As with all self-reported variables, data on physical activity and sports history may be affected by recall bias or limited precision. Segmental BIA estimates are influenced by underlying assumptions regarding body geometry and by the algorithms embedded in proprietary software, which may affect accuracy in individuals with atypical body composition. The use of a single commercial device also presents challenges for replication across systems employing different frequencies or electrode configurations. Finally, vendor-specific indices such as the Health Score, while effective within the current analytic context, are not standardized or fully transparent, which may limit reproducibility and transferability across platforms.

In conclusion, this study demonstrates that raw bioelectrical impedance analysis data, when interpreted through physiologically grounded and transparent machine learning models, can yield clinically meaningful classifications of osteopenia. The identification of predictors related to fluid distribution, body composition, and localized muscularity underscores the complex and multifaceted nature of bone health. These findings highlight the importance of BIA in identifying both systemic and regional factors that affect skeletal integrity. However, these conclusions must be interpreted cautiously in light of the study’s design and sample characteristics. Future research should focus on longitudinal validation, external replication, and the inclusion of direct mechanical loading data to refine the understanding of predictors of bone health.

## 5. Conclusions

This study supports the utility of explainable ML models in classifying osteopenia based on raw BIA outputs and physical activity variables. Among the tested classifiers, the NN achieved the highest cross-validated accuracy (92.12%) using 34 selected predictors, demonstrating that ML can effectively distinguish individuals with osteopenia. SHAP analysis identified influential BIA-derived features contributing to model performance, including the Edema Index, physical activity status, protein mass, hydration status, whole-body reactance at 50 kHz, and segmental lean tissue mass. By focusing on women aged 40 to 55, the study targeted the perimenopausal transition, a period characterized by early shifts in hydration status, tissue distribution, and bone remodeling. This biologically specific sampling frame facilitated the detection of alterations in BIA-based profiles associated with declining BMD, even in the absence of clinically overt osteoporosis. The model’s performance within this transitional age window underscores the promise of ML-based screening for early identification and timely intervention. These findings suggest that BIA-integrated, ML-guided screening could serve as a scalable and cost-effective adjunct to DXA, particularly in settings with limited access to imaging technologies. The integration of interpretable ML models into clinical or performance contexts may inform individualized strategies for exercise, nutrition, and the longitudinal monitoring of bone health. Continued refinement using larger, more diverse datasets and expanded physiological inputs may enhance predictive performance and broaden applicability.

## Figures and Tables

**Table 1:** Participant characteristics.

	Osteopenic (Mean ± SD, n)	Non-Osteopenic (Mean ± SD, n)	T-Score	*p* Value
Age	48.8 ± 3.9 (n = 33)	46.6 ± 4.5 (n = 105)	−2.69	0.009
Height (cm)	164.2 ± 7.5 (n = 33)	165.3 ± 5.9 (n = 104)	0.80	0.428
Weight (kg)	62.0 ± 7.9 (n = 33)	73.0 ± 13.8 (n = 105)	5.67	<0.001
BMI (kg/m^2^)	23.1 ± 3.5 (n = 33)	26.6 ± 4.8 (n = 105)	4.51	<0.001
Fat Mass (kg)	19.9 ± 7.1 (n = 33)	26.5 ± 10.4 (n = 103)	4.10	<0.001
% Body Fat	31.4 ± 7.8 (n = 33)	35.1 ± 8.6 (n = 103)	2.29	0.026
Lean Mass (kg)	39.6 ± 3.8 (n = 33)	43.6 ± 4.4 (n = 103)	5.04	<0.001

**Table 2:** Testing performance of ML classifiers.

Model	Accuracy	Recall	Precision	F1-Score	ROC AUC	Best Hyperparameters	Features
NNs	0.9212	0.7667	0.8914	0.8199	0.9311	{‘activation’: ‘relu’, ‘alpha’: 0.0001, ‘hidden_layer_sizes’: (10, 20, 50), ‘learning_rate’: ‘constant’, ‘solver’: ‘adam’}	34
LR	0.8701	0.4905	0.9500	0.6424	0.8626	{‘C’: 0.1, ‘solver’: ‘newton-cg’}	53
SVM	0.7611	0.0000	0.0000	0.0000	0.4749	{‘C’: 0.1, ‘kernel’: linear’}	1
XGBoost	0.8847	0.7333	0.7886	0.7547	0.8243	{‘learning_rate’: 0.1, ‘max_depth’: 5, ‘min_child_weight’: 1, ‘n_estimators’: 200}	13

**Table 3:** Most informative predictors for BMD prediction.

Predictors	Type of Predictor
Minerals	Numeric
Edema Index (EI)	Numeric
Total Body Water	Numeric
Right Leg Lean Index Score	Numeric
Mineral%	Numeric
Protein%	Numeric
20 kHz, Impedance (Z) of Left Arm	Numeric
Edema Index	Numeric
Activity Status	Categorical
Athlete Status	Categorical
Free Fat Mass	Numeric
50 kHz, Reactance (Xc) of Left Arm	Numeric
Fat Mass (FM)	Numeric
Protein Mass	Numeric
FM Normal Range Lower Limit	Numeric
Health Score	Numeric
Right Arm Lean Index Score	Numeric
Waist–Height Ratio (WHtR)	Numeric
Skeletal Muscle Index	Numeric
20 kHz, Impedance (Z) of Left Arm	Numeric
50 kHz, Impedance (Z) of Right Leg	Numeric
250 kHz, Impedance (Z) of Left Arm	Numeric
Protein Mass.1	Numeric
Body Mass Index (BMI)	Numeric
50 kHz, Impedance (Z) of Left Leg	Numeric
TBW/FFM.1	Numeric
Trunk Fat Mass.1	Numeric
50 kHz, Phase Angle of Right Arm	Numeric
Soft Lean Mass (SLM)	Numeric
50 kHz, Reactance (Xc) of Right Leg	Numeric
Right Leg Fat Mass	Numeric
5 kHz, Phase Angle of Right Arm	Numeric
Fat-Free Mass Index	Numeric
20 kHz, Impedance (Z) of Right Leg	Numeric

## Data Availability

The data used in this study are confidential and cannot be shared due to stringent primary regulations and ethical considerations. Access to the data is strictly restricted to the research team so that we can protect the participants’ identity and well-being.

## Supplementary Materials

The following supporting information can be downloaded at https://www.mdpi.com/article/10.3390/jfmk10030262/s1: Table S1: Variables used in the Machine Learning Model.
