# Supplementary material for: Interpretable Machine Learning for Osteopenia Detection: A Proof-of-Concept Study Using Bioelectrical Impedance in Perimenopausal Women
Source: J Funct Morphol Kinesiol. 2025 Jul 11;10(3):262. doi: 10.3390/jfmk10030262 (PMC12286257; doi:10.3390/jfmk10030262)
Supplement: Supplementary file 1 [file jfmk-10-00262-s001.zip › jfmk-3654547-supplementary.pdf]

Table S1: Variables used in the Machine Learning Model

Non-BIA Variables

This category includes variables that are either self-reported (interview-based) or obtained through direct measurement but not derived from bioelectrical impedance analysis.

- **Age**: Chronological age in years, obtained through self-report or official records.
- **Menopausal status**: Categorical variable indicating reproductive stage (premenopausal or postmenopausal), assessed via structured interview.
- **Physical activity level**: Ordinal self-reported variable reflecting habitual physical activity (active or not).
- **Athlete status**: Binary self-identified status (athlete vs. non-athlete), based on organized sport participation or training history.
- **Height**: Measured in centimeters using a stadiometer; not derived from BIA.

BIA Variables

1. Raw Bioelectrical Measurements: Fundamental electrical properties measured across multiple frequencies and body segments.

- Impedance (Z) at 5, 20, 50, 100, 250 kHz (Whole Body, Right Arm, Left Arm, Right Leg, Left Leg, Trunk)
- Phase Angle at 5 kHz and 50 kHz (Whole Body and Limbs)
- Reactance (Xc) at 50 kHz (Whole Body and Limbs)
- Resistance (R) at 50 kHz (Whole Body)
- BIVA R(Z), BIVA Xc(Z)

2. Body Composition Estimates: Whole-body values for major tissue compartments derived from impedance modeling.

- Fat Mass (FM)
- Fat-Free Mass (FFM)
- Soft Lean Mass (SLM)
- Skeletal Muscle Mass (SMM)
- Body Cell Mass (BCM)
- Protein Mass
- Mineral Mass
- Percentage Body Fat (PBF)

- Protein %
- Mineral %
- SMM %

3. Segmental Composition Metrics: Regional estimates of lean, fat, and tissue mass by body segment.

- Right/Left Arm Lean Mass
- Right/Left Leg Lean Mass
- Trunk Lean Mass
- Right/Left Arm Fat Mass
- Right/Left Leg Fat Mass
- Trunk Fat Mass
- Right/Left Arm Tissue Mass
- Right/Left Leg Tissue Mass
- Trunk Tissue Mass
- Lean_Score of Whole Body
- Right/Left Arm Lean Index Score
- Trunk Lean Index Score
- Right/Left Leg Lean Index Score

4. Water Compartments: Estimates of fluid distribution within and outside cells, with derived hydration ratios.

- Intracellular Water (ICW)
- Extracellular Water (ECW)
- Total Body Water (TBW)
- ICW %
- ECW %
- TBW %
- TBW/FFM
- Edema Index (EI)

5. Composition-Based Ratios and Indices: Size-normalized values for comparative assessment of tissue composition.

- Fat Mass Index
- Fat-Free Mass Index
- Skeletal Muscle Mass Index (SMI)
- Appendicular Skeletal Muscle Index (ASMI)

6. Functional and Metabolic Estimates: Predictive metrics related to metabolism and neuromuscular performance.

- Basal Metabolic Rate (BMR)
- Total Energy Expenditure (TEE)
- Right/Left Hand Grip Force (kg)
- Right/Left Hand Grip Force (N)
- Right/Left Handgrip Strength Lower Limit
- Right/Left Handgrip Strength Upper Limit

7. Composite Health and Risk Scores: Device-generated indicators summarizing health risk or muscular condition.

- Health Score
- Muscle Quality Score
- Visceral Fat Area
- Subcutaneous Fat Area
- Visceral Fat Area Level
